# The Relationship between the Burden of Acromegaly, Associated Comorbidities, Complications and Disease Status

**DOI:** 10.3390/jcm12196309

**Published:** 2023-09-30

**Authors:** Michał Elbaum, Marcin Kałużny, Aleksandra Jawiarczyk-Przybyłowska, Beata Wojtczak, Grzegorz Zieliński, Marek Bolanowski

**Affiliations:** 1 Department of Endocrinology, Diabetes and Isotope Therapy, Wroclaw Medical University, Wybrzeże L. Pasteura 4, 50-367 Wroclaw, Poland; 2 Department of General, Minimally Invasive and Endocrine Surgery, Wroclaw Medical University, Borowska Str. 213, 50-556 Wroclaw, Poland; 3 Department of Neurosurgery, Military Institute of Medicine—National Research Institute, 128 Szaserów Str., 04-141 Warsaw, Poland

**Keywords:** acromegaly, complications, comorbidities, therapy

## Abstract

Uncontrolled acromegaly causes increased morbidity and mortality. The analysis of acromegaly comorbidities and complications is important when establishing a standard of care for the entire population of acromegaly patients. The aim of this study was to determine the frequency of comorbidities and complications of acromegaly and their dependence on the activity of the disease. A retrospective analysis of medical records from 124 patients with acromegaly was carried out, including 39 who were cured, 73 treated with somatostatin analogs and 12 newly diagnosed patients. The incidence of comorbidities and complications was very high, and those most frequently observed were arterial hypertension, multinodular goiter, lipid disorders, hypopituitarism and degenerative changes. At least one complication of acromegaly was observed in 92% of patients undergoing successful neurosurgery and in all pharmacologically treated patients. By contrast, two or more complications were observed in 77% of cured patients and in pharmacologically controlled and uncontrolled patients, 82% and 91%, respectively. Conclusions: Acromegaly is associated with a high prevalence of complications. Active acromegaly is associated with a higher incidence of complications than in treated groups. Untreated patients have more complications than treated patients. Successfully cured patients have significantly fewer complications than pharmacologically controlled patients and patients with active acromegaly.

## 1. Introduction

Acromegaly is a rare, chronic disease that is caused, in most cases, by an over-secretion of growth hormones (GHs) from a benign pituitary tumor. The diagnosis of this disease is often delayed due to the slow progression of discrete symptomatology. Nevertheless, cardiovascular and respiratory complications, glycemic, respiratory, hormonal, metabolic and musculoskeletal disorders are already present at the time of diagnosis. When not treated, acromegaly reduces life expectancy by almost 10 years, leads to disabilities, disturbs professional employment and may decrease quality of life [1]. Only early diagnosis and effective treatment can give an opportunity to restore life expectancy and improve quality of life [2]. Premature mortality in acromegaly is mainly due to cardiovascular complications and respiratory system diseases. Moreover, there is an increased risk of various neoplasms [1,3,4,5,6,7]. Some comorbidities, such as hypertension and diabetes mellitus, may persist even after curing acromegaly successfully [8]. Over the last several years, life expectancy has increased due to more efficacious therapies for acromegaly and its comorbidities, and causes of mortality in acromegaly have changed toward neoplasm predominance, as seen in the general aging population [2,9].

The disease occurs equally in both sexes, with a slight prevalence in women. The mean age of diagnosis for acromegaly is situated between 40 and 50 years [3,10,11,12]. The time from the onset of symptoms to the final diagnosis has shortened (median < 5 years) recently [3,11,13,14]. This might be caused by a better awareness of the disease due to various educational efforts and the better availability of the health system. The frequency of acromegaly in patients with type 2 diabetes mellitus is greater than in the general population; there are an estimated 480 cases per million [15], whereas in patients with obstructive sleep apnea, there are 2000 cases per million [16]. There is no register of acromegaly patients in Poland yet.

The aim of this study was to assess the frequency of acromegaly comorbidities and complications in a group of patients from one academic center within the years 2011–2016 and relate them to the present status of the disease assessed using the current biochemical activity of the disease.

## 2. Materials and Methods

A retrospective analysis was carried out on the basis of 303 records taken from the archive of the University Clinical Hospital (known before as Independent Public Clinical Hospital No 1). Data included a total of 124 consecutive patients with confirmed acromegaly, out of which 74 women (59.7%) and 50 men (40.3%) were hospitalized in the Clinic of Endocrinology, Diabetes and Isotope Therapy in 2011–2016. The patients were divided into 4 groups: cured patients (A_CUR_), patients controlled by medical therapy (A_CON_), patients not controlled but with active disease (A_ACT_) and de novo patients with recently diagnosed acromegaly before any treatment (A_NOV_). The analysis did not include patients in whom acromegaly was not confirmed by the end of the study. We assessed the groups in terms of age, age at diagnosis and duration of disease since diagnosis. Basic characteristics of the patient groups are given in Table 1, and statistical calculations in Table A1.

Patients were divided according to criteria proposed by the Endocrine Society (2014), in which well-controlled patients had normal IGF-1 levels, expressed in proportion to the upper limit of the normal range (IGF-I/ULN) for sex and current age, and random serum was GH < 1 μg/L during medical therapy. Cured patients after successful neurosurgery did not require adjuvant medical therapy—their IGF-1 concentrations were normal—and GH was <1 μg/L after oral glucose load [17]. When hormonal normalization failed, these patients qualified for the group with active disease. In some cases, a discrepancy between GH and IGF-1 concentrations occurred. Then, we considered them, but the final group division was based on clinical status too. For example, in the group of 39 cured patients, 30 (77%) fulfilled both hormonal criteria of remission. Of the remaining 9 patients, 3 of them had normal IGF-1, but their GH was slightly elevated (1.09; 1.04; 1.22 μg/L). In six patients, we observed suppressed GH values (0.05–0.46 μg/L), but their IGF-1 values were between 113 and 150%. The patient with an IGF-1 value of 150% suppressed GH to 0.36 μg/L. Among the other five, three of them had IGF-1 values < 120% and two others had IGF-1 values below 130%. None of them presented clinical symptoms for the activity of acromegaly. On the other hand, in the group of 56 active patients, 49 (88%) presented elevated IGF-1 only and 7 (13%) did not show suppressed GH levels. In the latter subgroup, IGF-1 values varied between 91 and 102%. All of them presented symptoms of active acromegaly. Table A2 shows pairwise comparisons of groups for IGF, GH.

Patient information cards were analyzed in terms of identified complications. Complications diagnosed before the start of this study and complications that were diagnosed during this study were taken into account. During hospitalization, all patients underwent a wide range of hormonal tests. In accordance with the recommendations of the Polish Diabetes Association, screening tests for diabetes and prediabetes were performed. Criteria for the diagnosis of diabetes were glycemia > 125 mg/dL (≥7 mmol/L) found in fasting patients in two independent tests or the result of glycemia ≥ 200 mg/dL (≥11.1 mmol/L) in the 2nd hour of the oral glucose load test. Prediabetes included impaired fasting glycemia, defined as morning glucose between 100 and 125 mg/dL (5.6 to 6.9 mmol/L), and impaired glucose tolerance, defined as two-hour glucose levels of 140 to 199 mg/dL (7.8 to 11.0 mmol/L) during the 75 g oral glucose tolerance test. The glycated hemoglobin index used during this study is not recommended in Poland for diagnosing diabetes [18]. During this study, we measured blood pressure and lipid profiles. Patients underwent an electrocardiogram, an ultrasound diagnostic (abdomen and thyroid), an echocardiogram and a colonoscopy. Due to a lack of specific equipment in the clinic and hospital, patients were not tested for obstructive sleep apnea during hospitalizations.

This study had approval from the Bioethics Committee of Medical University in Wroclaw and approval from the Hospital Executive. This study is supported by a Research Grant from Ipsen, Poland.

Using the methods of descriptive statistics, basic data were compiled and broken down into groups: A_CUR_, A_CON_, A_ACT_ and A_NOV_. Data analyses were performed using the R statistical environment in version 3.6.2 and additional packages dedicated to specific types of data analysis. For each variable or group of variables, a basic statistical description was performed first, taking into account the calculation of the distribution’s skewness. Due to the high skewness of most of the analyzed distributions and the occurrence of outliers, it was decided that non-parametric statistical analyses should be used to answer the research questions.

## 3. Results

The study population of patients with acromegaly was 124. The A_ACT_ and A_CUR_ groups were the most numerous, with 56 and 39 patients, respectively. The population of individuals in the A_CON_ and A_NOV_ groups was 17 and 12, respectively.

The mean age of the entire group was 55.4 ± 14.39 years; for women, the mean age was 57.04 ± 14.99 years and 52.98 ± 13.4 years for men. We found no significant difference in age between the groups.

The mean age at diagnosis was 42.94 ± 14.38 years in the study population. Although the A_NOV_ group was older at the time of diagnosis, we found no statistically significant differences.

The average duration of disease from the time of diagnosis was 15.04 ± 10.13 years for the entire population. Patients in the A_NOV_ group had the shortest duration of acromegaly compared to the other groups (6.75 vs. 14.15 in A_CUR_, 17.35 in A_CON_ and 16.73 in A_ACT_).

Acromegaly coexisted with multiple endocrine tumors (MEN) in 4 patients, including MEN 1 in 3 patients and the McCune–Albright syndrome in one. Two patients with active acromegaly during the study period were treated with pasireotide in a clinical trial. One patient received oral octreotide in a clinical trial. A therapy combining a somatostatin analog and a dopamine agonist was used in two patients with active acromegaly. Five patients with acromegaly did not agree to surgical treatment. Table 2 shows the incidence of comorbidities and complications observed in the entire group of patients with acromegaly and their respective subgroups according to the division.

Table 3 shows the number of comorbidities or complications in each group. Out of 31 possible complications or comorbidities, at least one of them was diagnosed in 97.5% of patients. On the other hand, at least two of them were more frequent in the group of patients who were recently diagnosed (A_NOV_) or experiencing biochemically uncontrolled acromegaly (A_ACT_) and were less frequent in successfully treated patients (A_CUR_).

## 4. Discussion

The comorbidities and complications of acromegaly significantly increase morbidity and mortality in this disease and generate a huge cost of care for these patients. The most common include hypertension, goiter, hypopituitarism, lipid disorders, degenerative and deforming changes in the musculoskeletal system, as well as diabetes mellitus and cancer [19]. These complications require additional long-term treatment, significantly increasing the cost of treatment for patients with acromegaly. The analysis of acromegaly comorbidities and complications is important in the planning and management of a single patient, as well as in establishing a standard of care for the entire population of acromegaly patients. The described group is sufficiently large and representative, so the results obtained here may be useful in making diagnostic and therapeutic decisions for patients suffering from acromegaly.

In our patient groups, there was a predominance of women (59.7%), with the exception of the de novo group (41.67%). Surprisingly, de novo patients in our study were the oldest group with the highest age at diagnosis.

In our study, it was shown that, of the entire group of patients with acromegaly, the most common complications were hypertension (50.81%), thyroid goiter (49.19%) and lipid disorders (48.39%). They concerned about half of the respondents. In about one-third of the patients, hypopituitarism (35.48%), degenerative and deforming changes of the musculoskeletal system (33.06%) and cholelithiasis (32.26%) were found. The next most common complications in the whole group were diabetes mellitus and isolated adrenocorticotropic insufficiency (28.23% each), changes in echocardiogram and isolated thyrotropic insufficiency (18.55% each), non-pituitary tumors (16.94%), isolated gonadotropic insufficiency (16.13%), prediabetes and epilepsy (15.32% each), headaches (13.71%), kidney stones (12.1%), colorectal polyps (10.48%) and arrhythmias (8.87%). Obstructive sleep apnea was observed in only 4% of patients, which is much lower than that shown in other studies. This suggests that many of our patients were undiagnosed. 

In the Swedish population, the most common complication among patients with acromegaly is hypertension (48.6%), followed by the presence of tumors other than in the pituitary gland (43.7%), degenerative changes in the musculoskeletal system (32.9%), hypopituitarism (28.1%) and diabetes mellitus (17.6%) [20]. For comparison, in Pakistani patients, the most common complication was diabetes mellitus (39.32%), followed by hypertension (35.95%) [21]. An analysis of the Korean population based on the Korean Health Insurance Review and Assessment Claims database showed that the incidence of diabetes mellitus was higher than in the general population, 54.1% versus 15.1%, respectively. A similar situation was observed in the case of the diagnosis of heart failure, which amounted to 5.6% for patients with acromegaly compared to 2.6% in the general population [22].

An Israeli study, which analyzed the medical records of patients with acromegaly living in and around the city of Haifa and western Galilee, showed that among 77 patients, the most common complications were hypertension and diabetes mellitus at 45% and 44%, respectively. Osteoporosis, sleep apnea and carpal tunnel syndrome were reported in 17%, 17% and 9% of acromegalic patients, respectively. There were no statistically significant differences in the incidence of acromegaly and its complications between areas with low, medium and high levels of general air pollution [23].

Rolla and colleagues showed in a group of 179 patients whose data were analyzed over 42 years (1976–2018), a comparable incidence of hypertension and nodular goiter at 58% and 52%, respectively. The most common complications were lipid disorders, which were observed in 74% of patients [24]. The incidence of acromegaly complications was also assessed in a larger population of patients in a Polish multicenter study. Among 148 patients, the most common complications were thyroid goiter (52%) and hypertension (58%), followed by carbohydrate metabolism disorders, prediabetes (34%) and diabetes mellitus (33%), respectively. Obstructive sleep apnea syndrome occurred in 14% of patients, while non-pituitary tumors occurred in 9% [25].

Kamusheva et al. conducted a retrospective multicenter analysis of data from 191 patients with acromegaly from Bulgaria. The authors showed that more than 95% of patients had at least one complication. The most common complications were other endocrine diseases, metabolic diseases (96.7%), cardiovascular complications (70.7%) and musculoskeletal complications at 22%. Statistically, complications were more common in women and people over 60 years of age [26]. Our results indicate a similar incidence of at least one complication (97.5%). When assessing the occurrence of two or more complications, we showed that they occurred in 100% of patients with de novo-diagnosed acromegaly, 91% of patients with the uncontrolled disease, 82% of patients who achieved biochemical control of their disease during therapy with somatostatin analogs and in 77% of cured patients.

An American study based on data obtained from the IBM database showed a much higher incidence of acromegaly-related diseases than in the group of other patients. For cardiovascular diseases, it was 67.6% vs. 48.4%; for hypopituitarism and hypothalamic diseases, it was 26.3% vs. 0.2%; for sleep apnea, it was 24.9% vs. 7.8%; for cancer, it was 22.6% vs. 8.4%; for joint and muscle diseases, it was 19.9% vs. 12.7%; for type 2 diabetes mellitus, it was 19.0% vs. 8.9%; and for bone disease, it was 8.2% vs. 3.7%, respectively. All these differences were statistically significant [27]. Chuang showed, based on the analysis of another American database, that in the first year after neurosurgery compared to pharmacological treatment, patients were more often diagnosed with arterial hypertension (50.4% vs. 32.0%), sleep apnea syndrome (31.6% vs. 15.8%) and arrhythmias (16.7% vs. 7.0%), respectively [28]. Broder performed a database analysis of 2171 acromegaly patients in the U.S. Among these patients, 47.6% had diseases that increased the risk of cardiovascular complications, including 31% with hypertension, 19% with hypertriglyceridemia and 17.5% with diabetes mellitus. In addition, 25.6% had musculoskeletal problems, 16.6% had hypopituitarism, 11.5% had sleep apnea, 10.3% had cardiovascular complications and 6.6% had bowel cancer [29]. Yuen showed that patients with acromegaly, when compared to the control group, had a statistically significant higher risk of developing hypertension (40.4% vs. 13.6%), thyroid diseases (31.9% vs. 8.9%), lung diseases (31.9% vs. 17.4%), diabetes mellitus (31.9% vs. 8.3%), lipid disorders (27.7% vs. 16%) and osteoarthritis (19.1% vs. 3.7%) [30].

One should be aware that some of these complications might be iatrogenic (various forms of hypopituitarism, cholelithiasis, cardiac arrhythmias), but available medical data did not always allow for an unambiguous assessment of their etiology. Gallbladder stones are a common complication in patients treated with somatostatin analogs. Prencipe et al. showed the presence of gallstones in 58.9% of patients in a group of 91 patients. Most of the events occurred 5 years after the initiation of treatment with a somatostatin analog [31]. In our study, gallstones were present in 32% of patients. In the A_CON_ group, they occurred in 53% of patients, whereas the incidence in the A_CUR_ and A_ACT_ groups was similar at 28% and 34%, respectively. Hypopituitarism is most often caused by neurosurgical treatment. The likelihood of hypopituitarism is greatest following surgery for a somatotropic macroadenoma of the anterior pituitary. In our observations, we found a higher incidence of hypopituitarism in the A_CON_ and A_ACT_ groups compared to the A_CUR_ group. As in other publications, we were also unable to retrospectively distinguish between the hypopituitarism caused by neurosurgical treatment and that caused by the destructive effects of the pituitary tumor itself.

The incidence of comorbidities and complications varied according to the patient’s disease status. In the group of patients cured of acromegaly, lipid disorders were most frequently observed (in about half of patients), followed by thyroid goiter (38.46%), hypertension (35.9%), pituitary insufficiency (33.33%), cholelithiasis (28.21%), adrenocorticotropic insufficiency (25.64%), degenerative changes (17.95%), diabetes mellitus, prediabetes and changes in echocardiography (15.38% each). In the group of patients with pharmacologically controlled acromegaly, thyroid goiter, arterial hypertension and cholelithiasis were most common (52.94% each), followed by lipid disorders and degenerative changes (47.06%), hypopituitarism, diabetes mellitus and cancer other than pituitary (41.18%), adrenocorticotropic insufficiency (35.29%) and epilepsy (35.29%). In the group of patients with active acromegaly, thyroid goiter (58.93%) and arterial hypertension (57.14%) were the most common, followed by lipid disorders (48.21%), hypopituitarism (42.86%) and degenerative changes (41.07%). Diabetes mellitus was present in 35.71%, gallstones in 33.93%, adrenocorticotropic insufficiency in 32.14%, thyrotropic insufficiency in 26.79% and changes in echocardiography in one-fourth of patients. Additionally, gonadotropic hypofunction occurred in 21.43%, while epilepsy, prediabetes and headaches were 14.29% each.

Observations on the incidence of complications in patients diagnosed with de novo acromegaly are extremely important where the effect of applied therapies is not present with only the effect of the disease itself. In this group, the most common were arterial hypertension (66.67%), lipid disorders (41.67%), thyroid goiter and nephrolithiasis (33.33% each), degenerative changes (25.0%), followed by diabetes mellitus, changes in echocardiography, non-pituitary tumors, prediabetes, arrhythmias, colorectal polyps, carpal tunnel syndrome and sleep apnea (16.67% each). Gallbladder stones, isolated adrenocorticotropic insufficiency, headaches and osteoporosis were also observed in this group (8.33% each). Due to the small number of patients in the A_NOW_ and A_CON_ groups, observations concerning them should be treated with caution.

Because of the retrospective character of our study, the frequency of some complications may be biased. The diagnostics and preparation of medical documentation were performed by various doctors employed at the clinic. We observed differences in the number of tests ordered and the completeness of the data entered. To prevent such situations in the future, a procedure should be introduced to ensure data uniformity.

## 5. Conclusions

Acromegaly is associated with a significant increase in complications. Based on our data, we conclude that active acromegaly is associated with a higher incidence of complications than in the treated groups. Untreated patients have more complications than treated patients. Successfully cured patients have significantly fewer complications than pharmacologically controlled patients and patients with active acromegaly.

## Figures and Tables

**Table 1:** Characteristics of the groups studied.

		Group A_CUR_ (N = 39)	Group A_CON_ (N = 17)	Group A_ACT_ (N = 56)	Group A_NOV_ (N = 12)	Entire Group (N = 124)
Sex	Men	11	8	24	7	50
	Women	28	9	32	5	74
Age (years)	Mean ± SD	54.0 ± 14.68	56.88 ± 14.16	56.55 ± 15.06	52.5 ± 11.84	55.4 ± 14.40 (*p* = 0.75)
	Min	21	35	27	35	21
	Max	81	82	86	72	86
Age at diagnosis (years)	Mean ± SD	42.72 ± 15.32	41.53 ± 15.59	42.25 ± 14.09	48.92 ± 11.49	42.94 ± 14.39 (*p* = 0.45)
	Min	16	23	18	32	16
	Max	73	75	74	69	75
Duration of the disease (years)	Mean ± SD	14.15 ± 9.41	17.35 ± 9.03	16.73 ± 11.15	6.75 ± 2.99	15.04 ± 10.13 (*p* ≤ 0.01)
	Min	4	5	5	3	3
	Max	41	38	50	13	50

**Table 2:** Incidence and frequency of comorbidities and complications in patients with acromegaly.

Comorbidity/Complication	Entire Group		A_CUR_		A_CON_		A_ACT_		A_NOV_	
Arterial hypertension	63	51%	14	36%	9	53%	32	57%	8	67%
Goiter	61	49%	15	38%	9	53%	33	59%	4	33%
Lipid disorders	60	48%	20	51%	8	47%	27	48%	5	42%
Hypopituitarism	44	35%	13	33%	7	41%	24	43%	0	0%
Joints degeneration	41	33%	7	18%	8	47%	23	41%	3	25%
Cholelithiasis	40	32%	11	28%	9	53%	19	34%	1	8%
Diabetes mellitus	35	28%	6	15%	7	41%	20	36%	2	17%
ACTH deficiency	35	28%	10	26%	6	35%	18	32%	1	8%
TSH deficiency	23	19%	5	13%	3	18%	15	27%	0	0%
Changes in echocardiogram	23	19%	6	15%	1	6%	14	25%	2	17%
Neoplasm other than pituitary	21	17%	4	10%	7	41%	8	14%	2	17%
Gonadotropin deficiency	20	16%	5	13%	3	18%	12	21%	0	0%
Prediabetes	19	15%	6	15%	3	18%	8	14%	2	17%
Epilepsy	19	15%	5	13%	6	35%	8	14%	0	0%
Headache	17	14%	5	13%	4	24%	7	13%	1	8%
Nephrolithiasis	15	12%	4	10%	1	6%	6	11%	4	33%
Colonic polyps	13	10%	4	10%	3	18%	4	7%	2	17%
Arrhythmia	11	9%	1	3%	1	6%	7	13%	2	17%
Osteoporosis	11	9%	4	10%	1	6%	5	9%	1	8%
Visual field defects	9	7%	3	8%	1	6%	5	9%	0	0%
Mental disorders	7	6%	1	3%	1	6%	5	9%	0	0%
Carpal tunnel syndrome	7	6%	2	5%	0	0%	3	5%	2	17%
Ischemic heart disease	6	5%	1	3%	0	0%	5	9%	0	0%
Heart failure	5	4%	0	0%	1	6%	4	7%	0	0%
Obstructive sleep apnea	5	4%	1	3%	0	0%	2	4%	2	17%
Menstrual disorders *	3	4% *	0	0%	0	0%	3	9%	0	0%
Stroke	2	2%	0	0%	0	0%	2	4%	0	0%
Diabetes insipidus	2	2%	0	0%	0	0%	2	4%	0	0%
Sleep disorders	2	2%	0	0%	0	0%	2	4%	0	0%
Embolism	1	1%	0	0%	0	0%	1	2%	0	0%
Bronchiectasis	1	1%	0	0%	0	0%	1	2%	0	0%

* Calculated in the female population.

**Table 3:** Number of comorbidities and complications for acromegaly in respective groups of patients.

	Group A_CUR_	Group A_CON_	Group A_ACT_	Group A_NOV_	Entire Group
	(N = 39)	(N = 17)	(N = 56)	(N = 12)	(N = 124)
Number of comorbidities and complications ≥ 1 (%)	36 (92%)	17 (100%)	56 (100%)	12 (100%)	121 (97.5%)
Number of comorbidities and complications ≥ 2 (%)	30 (77%)	14 (82%)	51 (91%)	12 (100%)	107 (86.3%)

## Data Availability

The datasets used and/or analyzed during the current study are available from the corresponding author upon reasonable request.

## Abbreviations

GH	Growth hormone
IGF-1	Insulin-like growth factor 1
MEN	Multiple Endocrine Neoplasia

## Appendix A

**Table A1:** The results of the Kruskal–Wallis test and pairwise comparisons of groups for age, age at diagnosis and duration of the disease.

	Age (Years)			Age at the Diagnosis (Years)			Duration of the Disease (Years)		
	chi^2^	df	*p*	chi^2^	df	*p*	chi^2^	df	*p*
	1.17	3	0.75	2.61	3	0.45	15.28	3	<0.01
Group pairs	W	*p*		W	*p*		W	*p*	
A_CUR_-A_CON_	302.5	0.61		312	0.737		245	0.12	
A_CUR_-A_ACT_	1011	0.54		1069	0.86		938	0.24	
A_CUR_-A_NOV_	210	0.6		185.5	0.29		109	<0.01	
A_CON_-A_ACT_	474	0.98		460.5	0.84		417	0.44	
A_CON_-A_NOV_	82.5	0.4		67	0.12		21.5	<0.01	
A_ACT_-A_NOV_	279	0.36		234	0.1		122	<0.01	

**Table A2:** The results of the Kruskal–Wallis test and pairwise comparisons of groups for IGF, GH.

	IGF			GH		
	chi^2^	df	*p*	chi^2^	df	*p*
	78.75	3	<0.01	72.87	3	<0.01
Group pairs	W	*p*		W	*p*	
A_CUR_-A_CON_	226	0.06		179	<0.01	
A_CUR_-A_ACT_	150	<0.01		74	<0.01	
A_CUR_-A_NOV_	6	<0.01		6	<0.01	
A_CON_-A_ACT_	23	<0.01		151	<0.01	
A_CON_-A_NOV_	0	<0.01		24	<0.01	
A_ACT_-A_NOV_	194	0.02		305	0.62	
